# Recombinant ASF Live Attenuated Virus Strains as Experimental Vaccine Candidates

**DOI:** 10.3390/v14050878

**Published:** 2022-04-23

**Authors:** Douglas P. Gladue, Manuel V. Borca

**Affiliations:** Plum Island Animal Disease Center, Agricultural Research Service, U.S. Department of Agriculture, Greenport, NY 11944, USA

**Keywords:** ASFV, ASF, African swine fever, African swine fever vaccines

## Abstract

African swine fever (ASF) is causing a pandemic affecting swine in a large geographical area of the Eastern Hemisphere, from Central Europe to East and Southeast Asia, and recently in the Americas, the Dominican Republic and Haiti. The etiological agent, ASF virus (ASFV), infects both domestic and wild swine and produces a variety of clinical presentations depending on the virus strain and the genetics of the pigs infected. No commercial vaccines are currently available, although experimental recombinant live attenuated vaccine candidates have been shown to be efficacious in protecting animals against disease when challenged with homologous virulent strains. This review attempts to systematically provide an overview of all the live attenuated strains that have been shown to be experimental vaccine candidates. Moreover, it aims to analyze the development of these vaccine candidates, obtained by deleting specific genes or group of genes, and their efficacy in preventing virus infection and clinical disease after being challenged with virulent isolates. This report summarizes all the experimental vaccine strains that have shown promise against the contemporary pandemic strain of African swine fever.

## 1. Introduction

African swine fever virus (ASFV) is causing a disease pandemic that affects swine industries from Central Europe to Southeast Asia [1]. Recently, the disease has reappeared in the Dominican Republic and Haiti [2] after more than 50 years of absence from the Western hemisphere. ASFV is a structurally complex virus with a large DNA genome encoding more than 150 genes [1]. While recent research has focused on the development of ASF vaccines, no vaccine is commercially available, resulting in disease management based mainly on culling infected animals and restricting the transport of susceptible animals [1]. Different experimental approaches have been applied to developing efficacious vaccines that can protect susceptible members of the suidae family from virus infection or, at least, prevent clinical disease. The development of inactivated vaccines using different methods of inactivation, as well as a variety of immunological adjuvants, has always produced unsatisfactory results [3,4]. Similarly, the use of virus proteins produced and delivered using a variety of expression vectors has been relatively inefficient in providing protection [5].

The most promising vaccine candidates have been derived from live attenuated strains of ASFV. Pigs infected with live attenuated virus strains are usually protected from infection or disease caused by virus isolates that are genetically and antigenically related (homologous) to the immunizing virus. ASF live attenuated virus strains have been obtained following different approaches. Initially, naturally attenuated virus strains were isolated from infected animals in the field [6,7,8]. These strains had decreased virulence in domestic swine, which encouraged their use as potential vaccine strains. In the 1960s, Portugal utilized a naturally attenuated field isolate belonging to the genotype I as a widely used vaccine. This approach resulted in a high proportion of vaccinated animals developing chronic ASF lesions [9]. This problem of residual virulence is common to all the naturally attenuated strains that have been used as vaccine candidates. Despite their limitations as vaccine candidates, some of these naturally attenuated field isolates have been useful in the study of ASF vaccinology. These naturally attenuated viruses induced adequate levels of protection when challenged with homologous viruses, yet retained some residual virulence in a proportion of the immunized animals. Well-characterized Portuguese field isolates OUR T88/3 [10,11,12] and NH/P68 [13] and, more recently, the Georgia-derivative attenuated field isolate in Latvia (strain Lv17/WB/Rie1, belonging to genotype II) [14] are good examples of these types of attenuated ASF viruses. Attempts to decrease the residual virulence of these field-attenuated ASF viruses by eliminating genes associated with virulence were unsuccessful. The resulting recombinant viruses either retained their virulence [15,16] or they lost their protective efficacy [16]. Therefore, the use of naturally attenuated isolates as vaccine candidates must be cautiously considered at this point. 

An alternative approach in the development of ASF attenuated virus strains is the progressive adaptation of virulent field isolates to replicate in a variety of primary swine cell cultures or established cell lines. The process of adaptation is usually accompanied by a progressive loss of virulence when inoculated into domestic pigs. This has been reported during the attenuation of several field isolates [10,13,17]. A good compilation of efforts to produce attenuated virus strains using this approach was recently published by Sereda et al. [18]. This review summarizes the development of vaccine candidates derived from field isolates obtained from different origins. One major problem associated with the adaptation of ASFV to grow in cell cultures is the drastic genomic changes that occur during the adaptation process. As an example, the adaptation of the ASFV Georgia isolate to replicate in the established Vero cell line has been associated with the deletion of large areas of the virus genome [19]. In all cases, the identification of the specific deleted genes causing attenuation in swine remain unknown. 

The last methodological approach used to generate attenuated strains of ASFV is the genetic manipulation of highly virulent field isolates. This approach requires, as a prerequisite, basic knowledge of virulence genes, followed by the removal of these genes from the virus genome to achieve attenuation. This systematic review summarizes all the published information regarding the development of attenuated ASFV strains by genetic manipulation, and the status of experimental vaccine development. 

## 2. Initial Identification of Genetic Determinants of Virulence in ASFV

The involvement of specific ASF virus genes, or groups of genes, in the process of virus virulence and disease production in the natural hosts remained largely unknown until the 1990s. The development of a method to delete specific genes from virulent ASFV isolates made possible the identification of virus genes involved in virulence during the infection of domestic pigs; these results are summarized in Table 1. The ASFV gene NL-S (DP71L)—identified by its partial homology to the neurovirulence-associated gene of herpes simplex virus ICP34.5—when deleted from the ASFV E70 isolate (∆NL-S), drastically reduced virus virulence in domestic pigs [20]. Pigs intramuscularly (IM) inoculated with a 10^2^ or 10^3^ tissue-culture infectious dose (TCID50) of ASFV∆NL-S remained clinically normal, presenting a long viremia (2 to 3 weeks). These animals survived a 30-day post-vaccination (pv) challenge with 10^2^ TCID50 of the homologous virulent E70 isolate, without presenting any clinical signs associated with ASF.

This report constituted the first identification of a genetic determinant of virulence in ASFV and presented the possibility of developing a recombinant ASF attenuated virus strain using genetic manipulation. This pioneering work facilitated the rational development of live attenuated vaccine candidates for ASF. The identification and subsequent manipulation of a genetic determinant of virulence has become a standard method for the development of experimental live attenuated vaccine strains.

The next ASFV gene to be discovered as a determinant of virulence was the UK (DP96R) gene [21]. This gene, located immediately upstream of NL-S in the E70 isolate, is highly conserved across many ASFV isolates, but showed no homology to any other previously characterized virus gene. All the animals inoculated with 10^2^ TCID_50_ IM of an ASFV E70 lacking the UK gene (∆UK) survived infection, but presented with a transient period of fever and lethargy right after inoculation. Similarly to observations after deletion of the NL-S gene, deletion of the UK gene does not affect the ability of the ∆UK virus to replicate in swine macrophages. Much like the in vivo studies with the ∆NL-S virus, animals infected with the ∆UK virus had prolonged viremias (4 to 6 weeks) and survived the IM challenge (by day 42 pv) with 10^4^ TCID50 of the homologous virulent E70 isolate, remaining clinically normal.

Deletion of the thymidine kinase (TK) gene was demonstrated in the highly virulent Malawi isolate. Since deletion of the TK gene impedes the replication of ASFV in swine macrophages, a TK deletion mutant virus (∆TK) was developed using a Malawi strain partially adapted to grow in Vero cells, although still fully virulent in domestic pigs [22]. Although ∆TK efficiently grows in Vero cells, a decreased ability to replicate in swine macrophages was observed. Seventy-five percent of animals inoculated IM with 104 TCID50 of the ∆TK virus survived infection, presenting with a transient fever but no other clinical sign of ASF. Similarly, as seen with animals inoculated with the ∆UK or ∆NL-S viruses, after the IM challenge of the ∆TK-inoculated animals on day 53 pv with 10^4^ TCID_50_ of the Malawi isolate, all the animals remained clinically normal.

The contribution of this research group to the development of attenuated ASF viruses was closed with the identification of a novel determinant of virulence, the 9GL gene [23]. This previously uncharacterized gene shared nucleotide similarity with the yeast ERV1 gene, which is involved in oxidative phosphorylation and cell growth. The 9GL amino acid sequence is highly conserved across ASFV isolates, further suggesting an important functional role. A recombinant ASFV lacking the 9GL gene, ∆9GL, produced using the highly virulent isolate Malawi Lil-20/1, presented a decreased ability to replicate in swine macrophages and produced virus particles with an altered morphology [23]. Importantly, ∆9GL was completely attenuated when IM inoculated into pigs (10^2^ to 10^6^ TCID50), with animals displaying a transient mild rise in body temperature and a reduced viremia lasting 3 to 4 weeks. The ASFV challenge was performed at 42 days pv using 10^4^ TCID50 of parental highly virulent Malawi Lil-20/1 administrated IM; all animals were completely protected, with the exception of a transient rise in body temperature in animals inoculated with the lower ∆9GL dose. 

To summarize, these four genes (NL-S, UK, TK and 9GL) were the first to be identified as determinants of virulence; this is because, when deleted from the respective ASFV isolate, it resulted in a drastic reduction in the virulence of the parental virus in domestic swine. Importantly, all four attenuated strains (∆NL-S, ∆UK, ∆TK and ∆9GL) efficiently protected pigs from disease produced after the challenge with the corresponding parental virus. Interestingly, although these genes were conserved across isolates, deletion of the same gene from the genome of different ASFV strains did not always produce the same phenotype in terms of virulence attenuation and protection efficacy, as described in the next section.

The next single gene described that was involved in ASFV virulence was a previously uncharacterized gene mapped on the right end of the virus genome DP148R [24]. Deletion of this gene in the virulent ASFV isolate Benin 97/1, producing the BeninΔDP148R virus, does not affect replication in swine macrophage cultures, but significantly reduced virulence in domestic pigs. Animals IM inoculated with 10^3^ HAD_50_ of the BeninΔDP148R virus only developed a transient rise in body temperature without additional signs related to ASF. The virus remained in the blood for approximately 3 weeks after infection. After receiving a booster 21 days after the first dose, animals were challenged IM with 10^4^ HAD_50_ of the parental virulent virus 3 weeks after the booster. The challenged animals survived with only a transitory period of fever. Interestingly, deletion of the DP148R gene from the genome of the highly virulent field Chinese isolate HLJ/18 did not reduce virulence in domestic swine [25]. The recombinant virus HLJ/18-DP148R-del, lacking the DP148R gene, when IM inoculated at a dose of 10^3^ HAD_50_, showed a virulent phenotype indistinguishable from that of the parental virus. Similarly, deletion of the DP148R gene from the genome of the Georgia 2007 isolate did not attenuate the virus [26]. Animals IM inoculated with 10^3^ HAD_50_ of Georgia∆DP148R developed a fatal disease with severity and kinetics similar to those of the parental Georgia strain. These reports constitute an example of how deletion of the same gene in different ASFV strains may produce recombinant viruses with very different virulent phenotypes. 

**Table 1:** Summary of initial vaccines against ASFV.

Vaccine	Vaccine Backbone	Gene Deleted	Reference	Protection
ΔNL-S	E70	NL-S	[20]	Homologous
ΔUK	E70	UK	[21]	Homologous
ΔTK	Malawi	TK	[22]	Homologous
Δ9GL	Malawi	9GL	[23]	Homologous
BeninΔDP148R	Benin	DP148R	[24]	Homologous
Ba71ΔCD2	Ba71	CD2	[27]	Homologous, Georgia 2007

Deletion of the viral CD2 (EP402R) gene produced complete attenuation of the virulent strain BA71 in swine [27]. BA71ΔCD2 is a recombinant virus in which the EP402 gene is replaced with a Lac repressor cassette under the control of the ASFV early/late promoter pU104 and the ∆Gus reporter gene. Recombination and virus stock production were performed in COS-1 cells. Pigs IM infected with 10^4^ or 10^6^ PFU of BA71ΔCD2 remained clinically normal with just a transient rise in body temperature and, importantly, were protected when IM challenged 24 days later with 10^3^ HAD_50_ of the virulent BA71 virus. Remarkably, all pigs immunized with BA71ΔCD2 also survived the IM challenge with 20 LD of the heterologous Georgia 2007/1 isolate. Interestingly, deletion of the CD2 gene from other virus isolates has not been associated with a reduction in virulence as in the case of BA71ΔCD2. Deletion of the CD2 gene from isolates such as Malawi Lil-20/1 [28], Georgia 2007 [29] and CN/GS/2018 [25] did not result in any decrease in virus virulence. It is possible that specific genetic characteristics of the BA71 strain allowed the deletion of the CD2 gene to result in complete attenuation [27]. As later presented in this review, deletion of CD2 has been shown to further potentiate the loss of virulence in already semi-attenuated viruses [30,31].

## 3. Attempts to Develop an Attenuated ASFV-Georgia Strain Using Individual Gene Deletion

The 2007 outbreak of the disease in the Republic of Georgia re-energized research in the development of ASF vaccines. The genetic determinants of virulence (NL-S, UK, TK, and 9GL) initially described above were used as the first gene candidates for deletion from the parental ASFV Georgia isolate (Table 2). 

The deletion of the NL-S gene from the highly virulent ASFV Georgia2010 isolate (genotype II) did not produce a similar result [32] to when deletion was performed in the E70 virus (belonging to genotype I) [20]. Animals IM inoculated with 10^4^ HAD_50_ either died from acute ASF disease or developed a delayed and subclinical form of the disease. Similarly, deletion of the UK gene from the Georgia2010 isolate did not result in reduced virulence in domestic pigs [32] as it did in the E70 isolate [21]. Pigs receiving IM 10^4^ HAD50 of ASFV-G-∆UK developed a fatal and acute disease indistinguishable from that caused by the parental Georgia2010 virus. 

Deletion of the TK gene in a virulent strain of ASFV Georgia adapted to replicate in Vero cells produced a virus (ASFV-G/V-∆TK) that, when IM inoculated into pigs up to 10^6^ TCID_50_, did not induce ASF disease; however, interestingly, these animals were not protected when IM challenged with 10^3^ TCID_50_ of the virulent parental Georgia strain [33]. 

Although deletion of the 9GL gene from the ASFV isolate Pretoriuskop/96/4 (Pret4) produced similar attenuation results [34] as in the Malawi Lil-20/1 isolate [23], the same gene deletion in the genome of the highly virulent ASFV Georgia2007 isolate did not reproduce those results [35]. While animals IM inoculated with relatively low doses (up to 10^3^ HAD_50_) of ASFV-G-∆9GL remained clinically normal (and were protected against the challenge with the virulent parental virus), pigs receiving 10^4^ HAD developed a fatal acute disease. These results indicate that ASFV-G-∆9GL retains residual virulence that is not seen in Malawi Lil-20/1, which remained attenuated even when IM administered at high doses (10^6^ HAD) [23].

**Table 2:** Determinants of virulence that did not fully attenuate ASFV-G.

Gene Deleted	Fully Attenuated	Protection	Reference
NL-S	No	-	[32]
UK	No	-	[32]
TK	Yes	No	[33]
9GL	Low doses, higher doses lethal	Yes	[35]
DP148R	No	-	[26]
CD2	No	-	[28,29]

These results highlight phenotypic differences when evaluating attenuation and protective efficacy induced in diverse ASFV isolates through the deletion of the same gene, even in the case of those genes being highly conserved across strains. The molecular basis of these differences is not understood at this time, but needs to be considered in the development of attenuated virus strains using field isolates.

## 4. Identification of Novel Determinants of Virulence in the ASFV-Georgia Strain

The first successful attempt to develop a live attenuated vaccine candidate based on the Georgia 2007 isolate was deletion of the 9GL gene, as described above [35]. Deletion of the 9GL gene from the Georgia2007 isolate resulted in attenuation at low doses (less than 10^3^ HAD_50_), but produced residual virulence at a higher dose. Regardless of its residual virulence, when used at a sublethal dose (10^2^ or 10^3^ HAD_50_), ASFV-G-∆9GL induced protection against the IM challenge with 10^3^ HAD_50_ of the Georgia2007 isolate, both at 21 and 28 days pv. The challenged animals remained clinically normal with an absence of replication of the challenge virus (sterile immunity). ASFV-G-∆9GL was one of the first recombinant attenuated viruses reported to induce protection against the epidemiologically important Georgia isolate.

Another recently identified determinant of virulence is the previously uncharacterized I177L gene [36]. Deletion of the I177L gene from the genome of the Georgia2007 isolate resulted in a significant decrease in viral replication in cultures of swine macrophages. The recombinant virus lacking this gene, ASFV-G-∆I177L, had a drastic decrease in virulence compared to its parental virus strain. Animals receiving up to 10^6^ HAD_50_ IM remained clinically normal, including an absence of fever. Importantly, animals receiving a wide range of ASFV-G-∆I177L doses were completely protected against the IM challenge with 10^2^ HAD_50_ of Georgia2007 virus at 28 days pv, even those receiving as little as 10^2^ HAD_50_ of ASFV-G-∆I177L. Remarkably, the animals vaccinated with doses of 10^4^ HAD_50_ or higher of ASFV-G-∆I177L developed sterile immunity against the challenge virus. 

The efficacy of ASFV-G-∆I177L was further tested, using, as the challenge virus, a highly virulent field isolate from Vietnam, TTKN/ASFV/DN/2019 [37]. TTKN/ASFV/DN/2019 is a derivative of the Georgia 2007 isolate that has evolved in the field for at least 12 years. The results demonstrated that ASFV-G-∆I177L is able to induce protection against TTKN/ASFV/DN/2019 with a similar efficacy as observed against the Georgia2007 strain [37]. These experiments were conducted using European- and Vietnamese-origin pigs; no differences in protective efficacy were observed between pig breeds.

The effect of the administrative route was also assessed using ASFV-G-∆I177L, to determine its potential use as an oral vaccine. Animals oronasally inoculated with 10^6^ HAD_50_ of ASFV-G-∆I177L and IM challenged 28 days later with 10^2^ HAD_50_ of the virulent Georgia isolate were protected, with an absence of clinical disease associated with ASF [38].

A limitation of most of the experimental LAVs is the ability of the vaccine viruses to be produced in stable cell cultures. The exception is BA71∆CD2, which replicates in Cos-1 cells [27]; all other described LAVs require primary swine macrophage cultures. Swine macrophages have to be freshly isolated from the donor swine using a tedious process and face additional regulatory requirements to produce a vaccine. Recently, a derivative of ASFV-G-∆I177L was developed by adapting ASFV-G-∆I177L, to replicate in an established swine cell line (PIPEC cells), facilitating its replication at an industrial scale [39]. The adapted virus ASFV-G-∆I177L/∆LVR has a large deletion in the left variable region of its genome [39]. In challenge studies performed in domestic pigs, ASFV-G-∆I177L/∆LVR maintained the same level of attenuation, immunogenic characteristics, and protective efficacy as ASFV-G-∆I177L [39].

The ASFV gene MGF-110-9L, a previously uncharacterized gene located on the left side of the virus genome, was shown to be involved in disease production [40]. A recombinant virus (ASFV-∆9L) lacking the MGF-110-9L gene, derived from the highly virulent ASFV Chinese isolate CN/GS/2018 (a derivative of the Georgia isolate), had a decreased ability to replicate in primary swine macrophage cell cultures. Importantly, ASFV-∆9L presented a partial reduction in virulence compared with its parental virus, since 60% of animals IM inoculated with 10^1^ HAD_50_ of ASFV-∆9L survived the infection, with only a transient fever and low viremia titers. No data were presented regarding the ability of ASFV-∆9L to protect against the challenge with the virulent parental virus. 

A report analyzed the role of another gene member of the MGF cluster, MGF-505-7R, in ASFV virulence in pigs [41]. The manuscript described the role of the MGF-505-7R gene in the down-modulation of the innate immune response and the construction of a recombinant virus (ASFV-∆7R), based on the virulent field isolate CN/GS/2018, harboring the deletion of the MGF-505-7R gene. Pigs IM inoculated with 10^1^ HA_50_ of the recombinant virus had no clinical sings associated with the disease during the 3-week observation period. ASFV-∆7R replication in the blood and tissues of inoculated pigs was decreased in comparison with the parental ASFV.

Another gene involved in disease production is MGF360-9L [42]. It was shown that the MGF360-9L gene is involved in the down-regulation of interferon expression. ASFV-∆360-9L, a recombinant virus lacking MGF360-9L obtained from the virulent ASFV CN/GS/2018 strain, had a slight decrease in replication ability in swine macrophages and was partially attenuated in pigs. Twenty percent of animals IM infected with 1 HAD_50_ presented with a fatal disease, another 20% had a transient clinical disease, and the rest remained clinically normal. Viremias in all the ASFV-∆360-9L infected pigs were present for more than 2 weeks. 

Recently, the ASFV gene I267L has been shown to inhibit RNA Pol-III-RIG-I-mediated innate antiviral responses [43] and has a role in virus virulence. Pigs were inoculated IM with 10 HAD_50_ of ASFVΔI267L, a recombinant virus obtained by deleting I267L from the genome of the virulent Chinese field strain CN/GS/2018. Eighty percent of the pigs inoculated with the ASFVΔI267L virus exhibited mild clinical signs, surviving during the 3-week observation period; the remaining 20% of animals developed a protracted and fatal disease. All these animals experienced prolonged viremias in comparison with pigs infected with the parental virus. Interestingly, another group showed that deletion of I267L from the virulent Chinese field isolate SY18 genome produced a virus (SY18ΔI267L) that, when IM inoculated at doses of 10^2^ TCID_50_, caused a severe and lethal disease in all the animals [44]. Perhaps the different inoculation doses or genetic diversity of the parental viruses explains the differences between the attenuated phenotype described for ASFVΔI267L and the virulent one of SY18ΔI267L. Another gene involved in virulence is MGF-505-7R. Two reports indicated that deletion of the gene in the genome of the virulent field isolate CN/GS/2018 decreased virus virulence in domestic pigs. Animals IM inoculated with 10 HAD_50_ showed either just a transient rise in body temperature or a complete absence of clinical signs associated with ASF [41]. In these cases [40], no data were reported regarding the ability of these recombinant viruses to protect inoculated animals against the parental virus challenge. Additionally, it should be noted that a very low dose of the virus (1 to 10 HAD, depending on the case) was used to assess the attenuation of each of these four recombinant viruses.

It was recently reported that I226R, a previously uncharacterized ASFV gene located on the right end of the virus genome, was shown to be involved in disease production [45]. Deletion of the gene from the genome of the virulent Chinese field isolate SY18 produced a virus, SY18∆I226R, with a drastic reduction in virulence. Pigs inoculated IM with doses as high as 10^7^ TCID_50_ remained clinically normal and had reduced viremia. After the IM challenge with 10^4^ TCID_50_ of the parental strain at 21 days pv, all the challenged pigs survived with no development of ASF disease.

The ASFV gene A137R has also been reported to be involved in virus virulence [46]. A137R is conserved across virus isolates and is located in the central region of the virus genome. The removal of the gene from the highly virulent ASFV Georgia2010 genome produced a moderate decrease in the replication of the obtained virus (ASFV-G-∆A137R) in swine macrophage cultures. In addition, ASFV-G-∆A137R showed a significant attenuation of virus virulence in swine. Animals inoculated IM with 10^2^ HAD_50_ of ASFV-G-∆A137R remained clinically healthy during the 28-day observational period, with medium-to-high viremias. Importantly, all the ASFV-G-∆A137R-inoculated animals were protected when IM challenged with 10^2^ HAD_50_ of the virulent parental strain ASFV-G. No evidence of replication of the challenge virus was observed in the ASFV-G-∆A137R-inoculated animals.

Deletion of the ASFV gene E184L, located on the right end of the virus genome, is also involved in disease production by the Georgia2010 isolate [47]. Sixty percent of domestic pigs IM inoculated with 10^2^ HAD_50_ of a recombinant virus lacking the E184L gene (ASFV-G∆E184L) survived the infection with only a transient fever, while the remaining animals experienced a significantly delayed presentation of fatal disease. All the animals surviving ASFV-G∆E184L infection were protected when IM challenged with 10^2^ HAD_50_ of the parental Georgia isolate.

A summary of the determinants of virulence that attenuate ASFV-Georgia-derived viruses is in Table 3.

## 5. Development of Recombinant Viruses Harboring Multiple-Gene Deletions

Several groups have attempted the development of recombinant viruses including the deletion of more than one gene. Multiple-gene deletion has been used to produce an attenuation when there is no clear identification of individual target genes, or to enhance the level of attenuation of a virus already harboring deletions affecting virulence. The obtained results are not always as expected, since combining different gene deletions does not produce a predictable linear effect.

An example of attenuation of virulent ASFV through deletion of a group of genes is the production of an attenuated strain, ASFV-G-∆MGF, with removal of six genes belonging to the MGF360 and MGF505 groups (genes MGF505-1R, MGF360-12L, MGF360-13L, MGF360-14L, MGF505-2R and MGF505-3R) from the genome of the highly virulent Georgia2007 isolate [53]. These genes are located on the left side of the virus genome and have been associated with host range specificity and the down-modulation of the innate immune response [54,55]. In addition, this area of the genome has been found to be deleted in attenuated field isolates or viruses adapted to replication in cell lines [10,19,56,57]. ASFV-G-∆MGF replicates in primary swine macrophage cell cultures as efficiently as the parental virus. Pigs inoculated IM with up to 10^4^ HAD_50_ remained clinically healthy. Importantly, when animals receiving as little as 10^2^ HAD_50_ were challenged with the virulent parental Georgia2007 strain, no signs of the disease were observed, despite detection of the challenge virus. A recombinant virus (HLJ/18-6GD) harboring similar gene deletion was developed using the highly virulent Chinese field isolate HLJ/18 [25], confirming results obtained with ASFV-G-∆MGF. Animals inoculated IM with up to 10^4^ HAD_50_ remained clinically normal and were protected against the challenge with 200 Pig Lethal Dose 50 (PLD50). Reversion to virulence studies performed with the recombinant virus HLJ/18-6GD demonstrated the presence of clinical signs associated with the disease in one of the animals included in the passage 5 and 6 groups, prompting questions about the genetic stability of the recombinant virus [25]. No information was provided regarding the genetic modifications associated with the reversion to a virulent phenotype in the viruses isolated in those late passages [25]. The same research group presented an improved version of the HLJ/18-6GD virus by additionally deleting the CD2-like gene. The resulting virus, HLJ/18-7GD, presented the same protective efficacy as HLJ/18-6GD and remained phenotypically stable in reversion to virulence studies [25]. 

A recent report [32] further studied the deletion of individual genes located in this area of the genome on the production of virus attenuation. A series of recombinant viruses were developed, using the Georgia2007 isolate as a parental virus, using different combinations of gene deletions. The virulence of all the recombinant viruses was tested using IM inoculation of 10^4^ HAD_50_ and challenge experiments performed with Georgia 2007. The co-deletion of MGF360-12L, MGF360-13L, MGF360-14L and MGF505-1R reduced virus replication in macrophages and resulted in complete attenuation in pigs; 64% of animals were protected after the challenge. Simultaneous deletion of MGF360-13L, MGF360-14L, MGF505-2R and MGF505-3R did not reduce virus replication in macrophages, and produced a delayed but lethal disease in all the inoculated animals. Lastly, co-deletion of genes MGF360-12L and MGF505-1R reduced virus replication in macrophages and eliminated virulence in pigs; 34% of the pigs were protected when challenged with the Georgia isolate. These results suggest that the deletion of genes MGF360-12L and MGF505-1R attenuates Georgia2007, but this recombinant virus is unable to efficaciously protect against the challenge. None of the recombinant viruses developed in this report [58] were able to induce full protection in challenge experiments like ASFV-G-∆MGF [53] and HLJ/18-6GD [25], including a virus harboring nine-gene deletions (MGF360-9 to 14L and MGF505-1R to 4R). Remarkably, the same nine-gene deletion was able to fully attenuate the virulent ASFV Benin isolate and induce full protection against the challenge with the parental virulent virus [59]. Animals inoculated twice with the recombinant virus Benin∆MGF IM, at 0 and 25 days pv, with 10^2^ HAD_50_ and 10^4^ HAD_50_, respectively, only developed a transient fever. These animals were protected from the IM challenge at 6 weeks pv with 10^4^ HAD_50_ of the parental virus, with a complete absence of clinical signs associated with the disease. It should be mentioned that the only available study on long-lasting immunity induced by a recombinant vaccine candidate was performed with the Benin∆MGF virus [60]. Animals inoculated with 10^4^ HAD_50_ of the Benin∆MGF virus were not protected when IM challenged 130 days later with 10^4^ HAD_50_ of the virulent parental Benin 97/1 strain.

A novel recombinant virus, ASFV-∆QP509L/QP383R—produced by the deletion of two previously uncharacterized virus genes, QP509L and QP383R, from the genome of a highly virulent ASF virus CN/GS/2018 strain—was highly attenuated when IM inoculated (10^4^ HAD_50_) in swine [52]. Animals remained clinically normal during the 17-day observational period, with low viremia titers. However, ASFV-∆QP509L/QP383R did not induce protection against the IM challenge with 10^2^ HAD_50_ of the virulent parental virus.

The recombinant virus SY18∆L7-11, harboring the deletion of five genes (L7L–L11L) located on the right end of the virus genome of the virulent strain SY18, demonstrated a partially attenuated phenotype in pigs [50]. SY18∆L7-11 did not have altered replication in swine macrophages, and when IM inoculated in pigs at doses of 10^3^ or 10^6^ HAD_50_, 90% of the animals survived with only a transitory fever and mild disease. All the surviving animals were protected against the IM challenge with 10^3^ HAD_50_ of the parental SY18 virus, with a few animals experiencing a transient fever.

An excellent example of unexpected synergistic activity between genes individually unable to induce attenuation by themselves is the recombinant virus ASFV-SY18-∆CD2v/UK [49]. This virus is a double-gene-deleted mutant constructed from a highly virulent Chinese field strain, ASFV-SY18, where the CD2 and the UK gene have been removed. ASFV-SY18-∆CD2v/UK has not affected replication in primary porcine macrophage cultures, yet was attenuated in pigs. Animals receiving IM 10^4^ TCID_50_ did not develop clinical signs associated with ASF, except for a transient fever. After the IM challenge at 28 days pv with 10^4^ TCID_50_ of the parental virus, all the ASFV-SY18-∆CD2v/UK-inoculated pigs remained healthy. A similar recombinant virus, HLJ/18-9GL&UK, harboring the same double gene deletion in the same parental virus retained residual virulence, producing a lethal disease in 50% of the animals IM inoculated with 10^4^ TCID_50_ [25]. This is yet another example of the challenges in trying to reproduce results by deleting the same genes from a very similar parental virus. Subtle differences in the ways in which the constructs were produced may explain the different results obtained by these two groups.

As mentioned earlier, the effect of multiple-gene deletions from the genome of a virus to enhance attenuation is difficult to predict. When attempting to improve a vaccine candidate’s safety profile, these additional deletions often do not increase attenuation, and/or there is a loss in vaccine efficacy.

This scenario played out during an attempt to increase the attenuation of a vaccine candidate through the deletion of two virus genes involved in pathogenesis: CD2v (EP402R) and the C-type lectin-like (EP153R) genes from the genome of the vaccine candidate ASFV-G-∆9GL [32]. ASFV-G-∆9GL/∆CD2 and ASFV-G-∆9GL/∆CD2v/∆EP153R were developed, harboring two and three gene deletions, respectively, from the genome of the parental virus ASFV-G-∆9GL. ASFV-G-∆9GL/∆CD2/∆EP153R, but not ASFV-G-∆9GL/∆CD2, had a decreased ability to replicate in swine macrophages when compared with parental ASFV-G-∆9GL, and both recombinant viruses induced almost undetectable viremia levels when IM inoculated (10^3^ HAD_50_) into domestic pigs. Importantly, both failed to protect against the challenge with the parental virulent ASFV-Georgia. Therefore, the deletion of CD2-like and C-type lectin-like genes significantly decreased the protective potential of ASFV-G-∆9GL as a vaccine candidate.

The effects of the individual or combined deletion of the same genes, CD2 and EP153R, from the Benin∆DP148R vaccine candidate produced different effects [31] than what was observed with ASFV-G-∆9GL [32]. The deletion of EP402R dramatically reduced viremia levels in infected pigs, while inducing protection after the challenge. The additional deletion of EP153R (Benin∆DP148R ∆EP153R∆EP402R) further attenuated the virus; however, there was no viremia and decreased protection efficacy. The deletion of EP153R alone did not result in further virus attenuation, did not reduce virus persistence in blood, and did not reduce vaccine efficacy. 

Similarly, to improve the safety profile of BA71∆CD2, two double recombinant viruses were developed, deleting either the EP153R gene or the UK gene (DP96R), BA71∆CD2EP153R and BA71∆CD2DP96R, respectively [31]. Comparative studies in pigs demonstrated that both of these deletions from BA71∆CD2 decreased its vaccine efficacy and did not improve its safety. 

ASFV-G-∆9GL represents another example of the unpredictability of genetic manipulation involving deletions of multiple genes [35]. The 9GL gene deletion was combined with the 6 genes deleted from ASFV-G-∆MGF [51]; this resulted in a recombinant virus, ASFV-G-∆9GL/∆MGF, that had a decreased ability to replicate in primary swine macrophage cultures relative to that of ASFV-G and ASFV-G-∆MGF, but similar to that of ASFV-G-∆9GL. ASFV-G-∆9GL/∆MGF was attenuated when IM inoculated into swine, even at doses as high as 10^6^ HAD_50_. These animals did not present detectable levels of virus in blood at any time post-infection, and they were not protected against the challenge IM with 10^6^ HAD_50_ of the virulent parental ASFV-G isolate [51].

In summary, most attempts to increase the safety profile of ASFV-G-∆9GL by combining the deletion of the 9GL gene with additional deletions have been unsuccessful, as evidenced by the significantly decreased protective efficacy [30,51]. One successful attempt was the deletion of the UK gene. As described earlier, single-gene deletion of UK from the Georgia isolate does not decrease parental virus virulence [48], but a virus harboring deletions of 9GL and UK (ASFV-G-∆9GL/∆UK) presented a drastically more attenuated phenotype than the parental ASFV-G-∆9GL [48]. While animals IM inoculated with 10^4^ HAD_50_ of ASFV-G-∆9GL developed a fatal form of ASF, those IM inoculated with up to 10^6^ HAD_50_ of ASFV-G-∆9GL/∆UK remained clinically normal. ASFV-G-∆9GL/∆UK grows in swine macrophages with a decreased ability compared with the parental ASFV-G-∆9GL, but effectively protected pigs IM inoculated with either 10^4^ or 10^6^ HAD_50_ of the virus against the IM challenge with 10^3^ HAD_50_ of Georgia isolate.

These many studies show that the deletion of similar genes in different vaccine candidates may produce a variety of phenotypic changes, and this unpredictability can be extended to the addition of multiple-gene deletions to an already attenuated strain. This observation is not surprising, considering that much remains unknown about the role of the deleted genes in virus virulence. Developing and testing recombinant viruses harboring each particular combination of gene deletions appears to be the only accurate approach to assessing the potential effect of those genetic manipulations on virulence attenuation and protective efficacy.

## 6. Conclusions

More than 40 recombinant virus strains have been developed by deleting genes from ASFV isolates, many of which were single deletions resulting in no observable phenotype to virus virulence (Table 4); of these, only 14 deletions are associated with an attenuation of highly virulent field isolates. Some of these recombinant viruses have a complete loss of virulence even at relatively high doses. Importantly, most of these attenuated strains are able to induce protection against their virulent parental virus at the doses tested. With additional evaluation, several of these attenuated strains may have the potential to be developed into commercial vaccines. 

The majority of the published results support the use of genetic manipulation to produce recombinant viruses with potential use as vaccine candidates. The lack of extensive experimental data and results for many of these recombinant viruses impedes further consideration of their potential as vaccine candidates. Basic information is needed before a recombinant virus can be seriously evaluated as a vaccine candidate, including: the onset and duration of protective efficacy; genetic stability; the stability of the attenuation phenotype; minimal protection doses; the possibility of being used with alternative routes of inoculation (particularly their efficacy using oral administration), etc.

The analysis of the published information makes it apparent that there is heterogeneity in the methods employed to evaluate the attenuation and protective efficacy of each of the vaccine candidates (Table 5). Vaccine candidates are being used at a wide range of doses (from 1 to 10^6^ HAD_50_), using pigs of different ages, and challenge procedures are using different doses (from 10 to 10^5^ HAD_50_) performed at different times post-immunization. Theses variables make it difficult to conduct a comparative analysis of all the published vaccine candidates. The very low doses used to test attenuation by some laboratories raise questions about the presence of residual virulence of those viruses if they were tested at higher doses. Residual virulence at higher doses is a common outcome during these types of studies and is a major objectionable factor in a vaccine strain. Differences in the challenge dose used is another consideration when comparing vaccine efficacy between studies. Standardized ASF vaccine development protocols would make for more robust evaluation of virus attenuation and assessment of protective efficacy.

The residual virulence and stability of the attenuated phenotype are two concerns when utilizing live attenuated viruses as vaccine strains in field conditions. Many recent reports have described the development of novel vaccine strains, but most of these are not accompanied by additional studies evaluating their safety profiles. Basic safety considerations include an absence of general and local toxicity and detection of reversion to virulence. The recombinant viruses HLJ/18-6GD and HLJ/18-7GD are an exception, with safety data available for both viruses [25].

Another topic that needs more attention is the development of DIVA-capable vaccine candidates. Currently, only two reports discuss the identification of potential target genes to support the development of accompanying DIVA tests [43,47]. As has already been experienced, it is expected that the deletion of immunogenic genes to serve as DIVA targets may have a detrimental effect on the protective efficacy of the vaccine strain [47]. As several studies have already shown, the effects of deleting more than one gene are unpredictable and require a standard experimental approach to comprehensively understand [30,47,51].

Heterogeneous phenotypes produced by the deletion of a specific gene or group of genes from different virus isolates is another research area that requires further investigation [25,26,28,29,30,32,33,51]. Many research groups have observed these differences, and it is expected that the investigation of the molecular bases of these differences will aid in the development of safer attenuated virus strains.

Finally, it is important to continue the effort to systematically discover and characterize virus genes associated with virulence. The significant work performed by the scientific community in discovering novel determinants of virulence has resulted in the development of a relatively large set of attenuated virus strains, and in the characterization of many virus genes with previously unknown functions in virus virulence. This knowledge is the essential initial step required for the development of potential novel vaccine candidates.

## Figures and Tables

**Table 3:** Determinants of virulence that attenuate ASFV-Georgia-derived viruses.

Gene Deleted	Fully Attenuated	Homologous Protection	Reference
9GL	Low doses, higher doses lethal	Yes	[35]
9Gl, UK	Yes	Yes	[48]
A137	Yes, only low doses tested	Yes	[46]
CD2, UK	Yes	Yes	[49]
E184L	No	Surviving animals	[47]
I177L	Yes	Yes	[36,37,38]
I226R	Yes	Yes	[45]
I267L	No	-	[43,44]
L7L–L11L *	No	Surviving animals	[50]
MGF-110-9L	Partial at low doses	-	[40]
MGF360-9L	Partial at low doses	-	[42]
MGF-505-7R	Yes, only low doses tested	-	[41]
Multiple MGF ^#^	Yes	Yes	[25,51]
QP509L/QP383R	Yes	No	[52]

* L7L-L11L consists of genes L7L, L8L, L9R, L10L, L11L; ^#^ multiple MGF consists of a deletion of 6 MGF genes.

**Table 4:** Single deletions in ASFV that resulted in no phenotype.

Gene	Isolate	Reference
A224L (4CL)	Malawi	[61]
A859L	Georgia	[62]
C962R	Georgia	[63]
CD2	Georgia	[30]
E165R	Ba71V	[64]
E296R	Ba71V	[65]
H240R	HLJ/2018	[66]
I8L	Georgia	[67]
KP117R (p22)	Georgia	[68]
L11L	Malawi	[69]
L83L	Georgia	[70]
MGF360 13L-14L	Georgia	[71]
MGF100-1R	GZ201801	[72]
MGF110-1L	Georgia	[73]
MGF360-16R	Georgia	[74]
MGF-360-1L	Georgia	[75]
Q174L	Ba71V	[76]
X69R	Georgia	[77]

E165R, E296R, H240R and Q174L were not tested in swine.

**Table 5:** Genetic deletions in ASFV-Georgia viruses resulting in experimental vaccines.

Gene Deleted	Dose Tested with Full Attenuation	Homologous Protection	Challenge Route and Dose	Reference
9GL	10^2^, 10^3^	Yes	IM 10^2^	[35]
9GI, UK	10^2^, 10^4^, 10^6^	Yes	IM 10^3^	[48]
A137	10^4^, 10^7^	Yes	IM 10^2^	[46]
CD2, UK	10^4^	Yes	IM 10^2^	[49]
DI 177L/DLVR *	10^2^, 10^4^, 10^6^	Yes	IM 10^2^	[39]
I177L	10^2^, 10^4^, 10^6^	Yes	IM 10^2^	[36,37,38]
I1226R	10^2^	Yes	IM 10^4^	[45]
Multiple MGF *	10^2^, 10^4^	Yes	IM 10^3^	[25,51]

* multiple MGF consists of a deletion of 6 MGF genes.

## Data Availability

Not Applicable.
